# Effects of Pre-Session Video Observational Modeling on Emotional Intelligence and 9 m Shooting Performance in U14 Male Handball Players: A Randomized Controlled Trial

**DOI:** 10.3390/children13050655

**Published:** 2026-05-07

**Authors:** Amayra Tannoubi, Noura Ahmed, John Elvis Hagan, Medina Srem-Sai, Vlad Adrian Geantă, Fairouz Azaiez

**Affiliations:** 1 High Institute of Sport and Physical Education of Gafsa, University of Gafsa, Gafsa 2100, Tunisia; amayra.tannoubi@issepgf.ugaf.tn (A.T.); noura.ahmedhafa@gmail.com (N.A.); fairouz.azaiez@issepgf.ugaf.tn (F.A.); 2 Sports Performance Optimization Research Laboratory (LR09SEP01), National Center for Sports Medicine and Science (CNMSS), Tunis 1003, Tunisia; 3 Department of Health, Physical Education and Recreation, University of Cape Coast, Cape Coast PMB TF0494, Ghana; 4 Neurocognition and Action—Biomechanics Research Group, Faculty of Psychology and Sports Science, Bielefeld University, 33501 Bielefeld, Germany; 5 Department of Health, Physical Education, Recreation and Sports, University of Education, Winneba P.O. Box 25, Ghana; mssai@uew.edu.gh; 6 Department of Physical Education and Sport, Faculty of Physical Education and Sport, Aurel Vlaicu University of Arad, 310010 Arad, Romania

**Keywords:** emotional intelligence, youth sport, shooting performance, Arabic EIS, observational learning

## Abstract

**Highlights:**

**What are the main findings?**

- A 6-week pre-session video observational modeling (VOM) program significantly improved both emotional intelligence (all dimensions) and 9 m shooting performance in U14 handball players compared to a control group.
- Improvements in emotional intelligence were significantly associated with performance gains, and post-intervention EI accounted for up to 43.7% of the variance in shooting performance.

**What are the implications of the main findings?**

- Brief, low-cost pre-session video interventions can simultaneously enhance psychological (EI) and technical performance outcomes in youth sport training.
- Emotional intelligence was associated with skill outcomes in the present study, supporting further investigation of its role in coaching and youth athlete development programs.

**Abstract:**

Background/Objectives: Emotional intelligence (EI) is a modifiable psychological competency associated with athletic performance, yet controlled interventions targeting EI in youth sport remain scarce. Pre-session video observational modeling (VOM) is effective for motor skill acquisition, but its potential to enhance EI has not been examined in a randomized design. This study aimed to evaluate the effects of a 6-week pre-session VOM program on EI and 9 m shooting performance in U14 male handball players, and to examine whether changes in EI were associated with performance improvements. Methods: Thirty-three male U14 handball players (*M* = 13.2 ± 0.4 years) were randomly assigned to a VOM group (*n* = 17) or a control group (*n* = 16). Before each training session, the VOM group viewed a standardized 3–5 min video demonstrating the 9 m shooting technique, while both groups completed identical training. EI was assessed using the Arabic Emotional Intelligence Scale (A-EIS), and performance as successful shots per five attempts. Data were analyzed using mixed ANOVA, between-group comparisons of change scores, and linear regression. Outcome assessment was conducted by an evaluator blinded to group allocation. Results: Groups did not differ at baseline (*p* > 0.05). The VOM group improved significantly across all EI dimensions and performance (*p* ≤ 0.005, η^2^p ≥ 0.176), whereas the control group showed statistically significant declines in most EI variables (*p* ≤ 0.019, *d* ≥ 0.66). Between-group differences were significant for all outcomes, with large effect sizes (*d* = 0.90–3.08, 95% CI [0.32, 3.85]). Significant Group × Time interactions were observed across variables (*p* ≤ 0.015, ω^2^p = 0.078–0.539). Improvements in EI was significantly associated with performance gains (β = 0.517, *p* = 0.002), and post-intervention EI was significantly associated with performance variance at post-test (*R*^2^ = 0.437, *p* < 0.001). Conclusions: Pre-session VOM was associated with concurrent improvements in emotional intelligence and 9 m shooting performance in youth handball players, with findings requiring replication in larger and more diverse samples before generalizable conclusions can be drawn. The observed relationship between EI and performance suggests that emotional processes may contribute to skill acquisition. These findings support the inclusion of brief observational strategies in youth training programs, while requiring replication in broader samples.

## 1. Introduction

### 1.1. Emotional Intelligence in Sport

Emotional intelligence (EI) defined as the capacity to perceive, use, understand, and regulate emotions in oneself and others [1,2] has attracted substantial empirical attention in sport psychology over the past two decades. Theoretical models converge on a multidimensional structure comprising distinct but interrelated competencies: emotional perception, facilitation of thought, understanding of emotional dynamics, and emotion regulation [3]. In sport-specific contexts, Lane et al. [4] operationalized this framework through the Emotional Intelligence Scale (EIS), a validated five-factor instrument measuring Using Emotions (UE), Appraisal of Own Emotions (AOW), Regulation of Emotions (RE), Social Skills (SK), and Appraisal of Others’ Emotions (AOT) each theoretically linked to distinct aspects of athletic functioning [4].

Empirical evidence consistently associates higher EI with more adaptive pre-competitive emotional profiles, including lower anxiety, greater vigor, and reduced confusion [5,6,7]. These affective advantages translate into measurable performance benefits: athletes with higher EI demonstrate superior attentional control under pressure, more effective coping strategies in adversity, and stronger interpersonal coordination in collective sport contexts [5,8].

From a mechanistic perspective, these benefits are likely mediated by enhanced emotion regulation capacities, which support attentional stability, decision-making efficiency, and motor execution under stress—core determinants of performance in dynamic sport environments [9,10]. Critically, EI is not a fixed dispositional trait: longitudinal and experimental evidence confirms that EI competencies respond to structured behavioral training [11], rendering EI a viable and practically meaningful target for sport psychology intervention.

### 1.2. Emotional Intelligence in Handball

Handball constitutes a particularly demanding environment for EI [12]. As a high-intensity contact sport characterized by rapid transitions, frequent adversity events (missed shots, fouls, counterattacks), and constant interpersonal coordination, handball places extraordinary demands on emotional regulation, appraisal of others’ emotional states, and the adaptive use of arousal for performance facilitation [13,14].

Research specifically examining EI in handball reports meaningful associations between EI and playing ability [15,16], EI and psychological well-being in elite players [17,18], and between EI dimensions and mood regulation in competitive populations [5]. However, no prior study has implemented a controlled EI-targeted intervention in youth handball players, a critical gap, given that early adolescence constitutes the most sensitive developmental window for EI skill acquisition [19,20] and that novice athletes are especially vulnerable to emotional dysregulation during initial skill-learning phases [21]. Importantly, this limitation constrains both theoretical advancement and applied practice, as it remains unclear whether EI can be systematically developed within ecologically valid training environments in youth handball populations.

### 1.3. Observational Learning and Video Modeling in Sport

Observational learning acquiring skills and behavioral schemas through systematic observation of a performing model represents one of the most fundamental mechanisms of human motor and psychological development [22,23].

In sport, video-based modeling has been operationalized as a structured pre-practice intervention in which athletes watch footage of a demonstrator performing the target skill before physical practice [24,25,26]. This format leverages the well-documented observation–practice coupling effect: watching a model immediately before practice activates relevant motor representations, directs attention toward the most informative kinematic cues, and reduces the cognitive load of early-stage skill execution [27,28,29,30]. Meta-analytic evidence supports video modeling’s efficacy for motor skill acquisition across sport contexts, with effect sizes ranging from moderate to large [31,32,33,34]. A key theoretical insight from this literature is that model identity is not the primary determinant of learning outcomes: the functional relevance of the demonstrated movement to the observer’s developmental stage is the critical variable [35,36]. Observational learning can effectively convey essential kinematic and behavioral information required for motor skill acquisition, provided that the demonstration is representative of the target skill and highlights key task features [30,37].

From a performance perspective, these processes are particularly relevant, as emotion regulation directly influences attentional control, decision-making speed, and motor execution under pressure key determinants of success in fast-paced team sports such as handball [38]. Beyond motor outcomes, the theoretical scope of observational learning extends to psychological and emotional dimensions. Social cognitive theory posits that observers encode not only the model’s motor actions but also the model’s affective responses to success and failure forming cognitive–affective scripts that guide subsequent behavior [39]. When athletes observe a demonstrator navigate high-demand performance situations managing missed shots, sustaining effort, and modulating arousal—they are simultaneously exposed to emotional behavioral scripts that may be internalized and reproduced during live practice [23]. This mechanism renders pre-session video modeling a theoretically plausible, though empirically untested, vehicle for the progressive consolidation of EI competencies, particularly emotion regulation and appraisal processes. Critically, however, it must be acknowledged that the present intervention is primarily technical in design: the video displayed shooting technique, not a dedicated emotional regulation protocol. Whether the incidental emotional content embedded in technical footage is sufficient to drive genuine EI development as distinct from motivational or attentional effects remains an open empirical question that the present study begins to address.

To the best of current knowledge, no previous controlled study has experimentally examined whether video-based observational learning can simultaneously affect both emotional intelligence and motor performance within a single sport intervention.

### 1.4. The Present Study

The present randomized controlled trial addresses this gap by integrating observational learning theory with emotional intelligence research in a youth sport context. Specifically, the study examines whether a structured 6-week pre-session video observational modeling program using footage of an anonymous athlete demonstrating the 9 m shooting technique can simultaneously improve EI and shooting performance in U14 male handball players, over and above identical training received by both groups. Three pre-registered hypotheses are tested:

**H1.**  *The VOM group showed significantly greater gains in EI total score and all five subscales compared to CG.*

**H2.**  *Mixed ANOVA revealed significant Group × Time interactions across all outcomes.*

**H3.**  *EI gains significantly predicted shooting performance gains, constituting preliminary correlational evidence consistent with a co-development hypothesis between EI and performance outcomes.*

## 2. Materials and Methods

### 2.1. Study Design and Registration

A parallel-group, randomized controlled trial was conducted between [October 2025] and [December 2025]. The trial was pre-registered on the Open Science Framework (OSF; https://osf.io/by53z) on 10 April 2026, following data collection and prior to any inferential statistical analysis; it therefore does not constitute a prospective pre-registration, which represents a recognized limitation of the present study. Reporting follows CONSORT 2010 guidelines [40] and the methodological framework recommended for sports performance research [41].

### 2.2. Ethical Statements

Ethical approval was obtained from the Local Ethics Committee affiliated with the High Institute of Sport and Physical Education of Gafsa, University of Gafsa, Tunisia (Protocol No. [ISSEP-GAFSA/EC/2025/60], dated 18 September 2025). The study protocols complied with the latest ethical standards set out in the Declarations of Helsinki [42]. The study was further conducted in accordance with the ethical standards in sport and exercise science research outlined by Harris et al. [43]. All participants and legal guardians provided written informed consent and assent.

### 2.3. Generative AI Disclosure

The authors used generative artificial intelligence tools for linguistic refinement and structural editing of the manuscript. The authors, who assume full responsibility for the content of this article, conducted all conceptual development, theoretical argumentation, and final editorial decisions.

### 2.4. Participants

Thirty-three male handball players from a Tunisian youth academy competing at the U14 national level (*M* = 13.2 years, *SD* = 0.4; height: *M* = 163.1 cm, *SD* = 2.8; weight: *M* = 53.9 kg, *SD* = 2.7; registered playing experience: *M* = 1.4 years, *SD* = 0.5) participated in this study. Inclusion criteria were: (1) novice competitive level (≤2 years of registered experience); (2) absence of musculoskeletal injury at the time of testing; and (3) written informed consent obtained from parents or legal guardians. In addition, all participants provided their assent to participate in the study. The exclusion criterion was the absence from more than one training session. No participants withdrew during the study, resulting in complete data for all 33 participants (retention rate = 100%). No missing data were observed; therefore, no imputation procedures were required.

**Figure 1:**
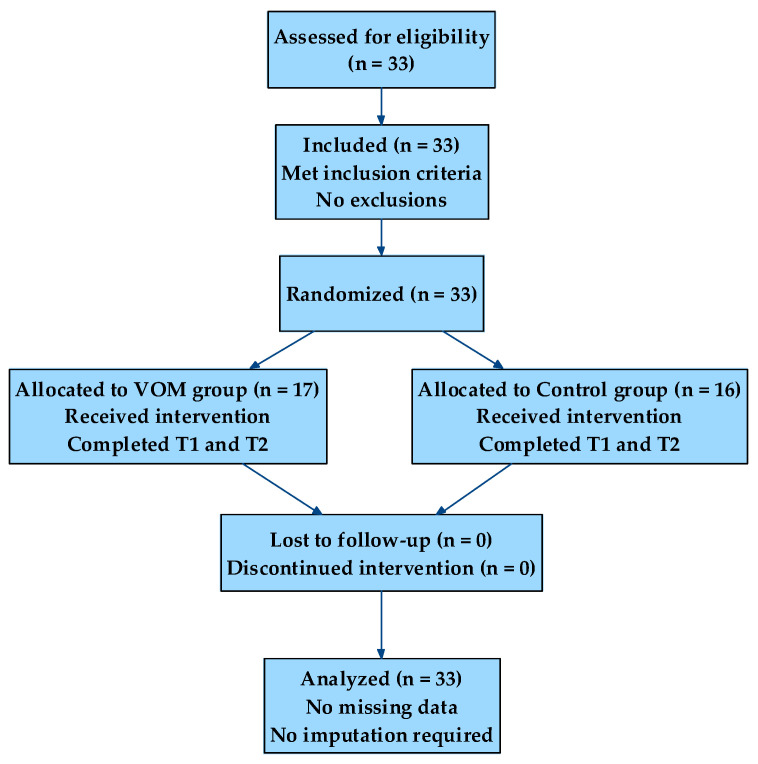
Flow diagram of participant progression through the study, including enrollment, randomization, allocation, follow-up, and analysis.

Participants were randomly assigned to groups using a stratified block randomization procedure based on baseline Performance/5 scores, with the allocation sequence generated by computer and concealed until completion of the T1 assessment. Allocation concealment was ensured using sealed, opaque envelopes prepared by an independent researcher not involved in data collection or analysis. Outcome assessment was conducted by an evaluator blinded to group allocation. An a priori power analysis conducted using G*Power 3.1 [44] for a Group × Time interaction in a 2 × 2 mixed ANOVA, assuming a medium-to-large effect size (η^2^p = 0.14), α = 0.05, and power (1 − β) = 0.80, indicated a minimum required sample size of *N* = 28. The final sample (*N* = 33) exceeded this requirement. The flow of participants throughout the study is presented in Figure 1.

### 2.5. Measures

#### 2.5.1. Emotional Intelligence—Arabic Emotional Intelligence Scale (A-EIS)

EI was assessed using the Arabic Emotional Intelligence Scale (A-EIS) [45], a rigorously validated cultural adaptation from the original scale of Lane et al. [4]. The A-EIS comprises 20 items distributed across five subscales: Using Emotions (UE; 4 items), Appraisal of Own Emotions (AOW; 4 items), Regulation of Emotions (RE; 3 items), Social Skills (SK; 4 items), and Appraisal of Others’ Emotions (AOT; 5 items), rated on a 5-point Likert scale (1 = strongly disagree to 5 = strongly agree; total range: 20–100).

Yahyaoui et al. [45] demonstrated satisfactory internal consistency (Cronbach’s α = 0.72–0.87 across subscales), acceptable confirmatory factor structure, and full measurement invariance across gender and sport type in Arabic-speaking populations. In the present sample, internal consistency was acceptable to high across subscales (Cronbach’s α = 0.78 to 0.88), supporting the reliability of the A-EIS in this cohort. It should be noted that the A-EIS was originally validated in adult physical education and sport populations; its use with 13-year-old athletes is supported by the instrument’s straightforward Likert-format items and age-appropriate language.

#### 2.5.2. Shooting Performance (Perf/5)

Shooting performance was assessed using a standardized 9 m penalty throw protocol: five attempts per participant under standardized conditions (same goal, goalkeeper, examiner, and court markings). Performance was operationalized as the number of successful shots per five trials (Perf/5; range: 0–5). The use of five attempts per assessment session aligns with standardized assessment procedures in published youth handball research [46,47].

### 2.6. Intervention

The intervention spanned 6 weeks (18 sessions, 3/week, ~90 min each). Both groups participated in identical handball training sessions delivered by the same coach, with equivalent duration, physical load, and technical content throughout. The sole methodological difference was the following pre-session component:

- VOM group: Pre-Session Video Observational Modeling:

Immediately before each training session, VOM participants collectively watched a 3–5 min standardized video featuring an anonymous athlete demonstrating the 9 m handball shooting technique. The model’s identity was unknown to all participants. Video content was selected to display: (1) correct and progressive technical execution of the shooting gesture across multiple repetitions; (2) realistic performance conditions (standard court, goal, and goalkeeper); and (3) successful shot outcomes as the terminal event of each sequence. The video content was standardized and remained identical across all 18 sessions. Beyond its technical content, the video was specifically selected to include scenes in which the athlete visibly maintained composure following missed attempts, sustained concentrated effort across successive repetitions, and modulated arousal levels throughout the sequence. This affective behavioral content embedded within, rather than separate from, the technical demonstration is theorized to provide observable emotional behavioral scripts that participants may internalize and apply during live practice. It is acknowledged, however, that the emotional content of the video was not formally coded or quantified, and that participants’ attentional focus (technical vs. emotional cues) was not assessed during viewing. No post-viewing debriefs, structured discussion, or additional instruction followed the viewing; participants proceeded directly to the training session.

The VOM sessions were conducted under standardized and controlled conditions to ensure optimal perceptual and attentional engagement. Video demonstrations were presented on a Lenovo laptop computer (15.6-inch screen; resolution 1920 × 1080 pixels) positioned approximately 1.5–2.0 m from the participants, ensuring clear visibility for all players [24]. Sessions took place in a quiet, enclosed room to minimize external distractions and auditory interference. Participants were seated and instructed to pay careful attention to the movements demonstrated without engaging in concurrent tasks. All videos were presented under identical environmental and viewing conditions across sessions. In addition, the viewing angle was standardized, and ambient lighting conditions were kept constant across sessions.

Intervention fidelity was monitored by the coach, who verified participant attendance and visual engagement during each video session. All VOM participants completed the full viewing protocol across the 18 sessions.

- Control group: Standard Training Only:

CG participants began each session directly, without any pre-session video viewing or supplementary activity. Both groups received identical coaching, technical feedback, and drill sequences throughout all sessions.

This within-session equivalence design ensures that any observed group differences are primarily attributable to the pre-session video modeling component within the constraints of the study design, and not to differences in training content, volume, or coaching quality.

### 2.7. Statistical Analyses

All analyses were conducted in R (version 4.4.0; [48] with a two-tailed significance threshold set at α = 0.05. The analytical procedure comprised four sequential stages. Assumption verification was performed by screening outliers using standardized z-scores (|z| > 3.29); analyses conducted with and without outliers yielded identical conclusions, and all observations were therefore retained. Normality was assessed using Shapiro–Wilk tests [49] within each Group × Time cell, and homogeneity of variances was evaluated using Levene’s test [50]. Shapiro–Wilk tests confirmed approximate normality in all Group × Time cells for UE, AOW, SK, AOT, IE Total, and Perf/5 (*p* > 0.05); RE in the CG at pre-test showed marginal deviation (*p* = 0.048), for which Wilcoxon robustness checks were specifically prioritized. All assumptions for parametric testing were verified prior to analysis.

Within-group changes were examined using paired *t*-tests to assess pre–post (T1–T2) differences within each group (VOM: *df* = 16; CG: *df* = 15), with Wilcoxon signed-rank tests conducted as non-parametric robustness checks [51]; effect sizes were calculated using paired Cohen’s *d* (Δ*M*/*SD*) [52].

Between-group comparisons were conducted using independent *t*-tests on gain scores (Δ = T2 − T1; *df* = 31), corroborated by Mann–Whitney U tests (effect size: rank-biserial r) [53], and a mixed-design ANOVA (Group [between-subjects] × Time [within-subjects]) was used to examine interaction effects for each outcome variable; both partial eta squared (η^2^p) and omega squared (ω^2^p) were reported, with the latter preferred for small samples (*n* < 50) due to reduced bias [54], and interpreted according to Cohen [52] thresholds (small ≥ 0.01, medium ≥ 0.06, large ≥ 0.14).

Correlational and predictive analyses were conducted using Pearson (*r*) and Spearman (ρ) correlations across all gain scores (*N* = 33). Two simple linear regression models were then tested: Model A examined whether changes in total EI predicted changes in performance (ΔEI total predicting Δperformance/5), while Model B assessed whether total EI at T2 predicted performance at T2. Multiple regression was not performed due to substantial multicollinearity among EI subscales in preliminary analyses (VIF = 10.4–25.5). In contrast, the retained models showed no evidence of multicollinearity (VIF = 1.00). Model fit was evaluated using *R*^2^, adjusted *R*^2^, and Cohen’s *f*^2^ (large ≥ 0.35) [52]. Given the confirmatory and pre-registered nature of the primary hypotheses (H1–H3) and the exploratory status of secondary analyses, no correction for multiple comparisons was applied across the seven outcome variables. Secondary outcomes should therefore be interpreted with appropriate caution and treated as hypothesis-generating.

## 3. Results

### 3.1. Baseline Equivalence

Randomization produced two anthropometrically equivalent groups (Table 1). Independent *t*-tests confirmed no significant pre-intervention differences on height [*t*(31) = 0.40, *p* = 0.692, *d* = 0.14] or weight [*t*(31) = 0.05, *p* = 0.961, *d* = 0.02], nor on any of the seven outcome variables (all *t* values < 1.50, all *p* values > 0.143; see Table 1), ruling out pre-existing group differences as alternative explanations for subsequent findings. No extreme outliers influencing the results were identified, and assumption checks supported the use of parametric analyses.

### 3.2. Descriptive Statistics

Descriptive statistics are presented in Table 2. At pre-test, both groups showed comparable EI and performance scores across all variables, with overlapping 95% confidence intervals. At post-test, a clear divergence between groups was observed: the VOM group reached *M* = 77.81 (*SD* = 1.48) on IE Total—with lower variability at post-test, possibly reflecting convergence toward higher scores or a ceiling effect of the instrument (A-EIS maximum = 100; VOM group post-test *M* = 77.81, *SD* = 1.48; 14 of 17 participants scored ≥ 76/100, representing 76% of the scale maximum, with minimal inter-individual variance—while the CG regressed to *M* = 35.72 (*SD* = 11.70), a decrease of 16.59 points from baseline. This compression of post-test scores should therefore be interpreted cautiously, as it may reflect not only improvement but also reduced score dispersion arising from measurement artifact or response tendencies. Shooting performance followed a similar pattern: VOM improved by 1.82 points (from 2.53 to 4.35/5), whereas CG showed a marginal, non-significant gain of 0.53 points.

### 3.3. Within-Group Evolution Paired T-Tests

Paired *t*-tests (Table 3) revealed that the VOM group improved significantly on all seven outcomes (*p* ≤ 0.005), with large-to-very-large effect sizes (*d* = 0.80–1.74). The greatest gains were observed for IE Total (Δ*M* = +24.88, *d* = 1.74), AOT (Δ*M* = +7.08, *d* = 1.62), and UE (Δ*M* = +6.06, *d* = 1.63). In contrast, the CG regressed significantly on five of seven variables (*p* ≤ 0.019), most markedly on RE (Δ*M* = −3.69, *d* = −2.17) and IE Total (Δ*M* = −16.59, *d* = −1.08). Only AOT (*p* = 0.172) and Performance (*p* = 0.141) did not change significantly in CG. All Wilcoxon signed-rank equivalents confirmed these findings.

### 3.4. Between-Group Gain Comparisons

Between-group comparisons of gain scores (Table 4) revealed significant VOM superiority across all seven outcomes (*p* ≤ 0.015). Effect sizes were universally large to very large (*d* = 0.90–3.08), with the largest differences observed for UE (*d* = 3.08), IE Total (*d* = 2.80), and RE (*d* = 2.51). Non-parametric Mann–Whitney *U* tests corroborated all findings; notably, the rank-biserial coefficient for RE reached *r* = −1.000, indicating complete separation between groups on this dimension. The systematic concordance between parametric and non-parametric *p*-values across all tests confirms that results are robust to distributional assumptions. These between-group effect size estimates should nevertheless be interpreted conservatively, given the small sample, the bidirectional group divergence, and the design characteristics.

### 3.5. Mixed ANOVA—Group × Time Interactions

Results of the mixed ANOVA are presented in Table 5. The Group × Time interaction—the primary inferential test—was statistically significant for all seven outcome variables (*F* ≥ 6.61, *p* ≤ 0.015), supporting H2. Interaction effects were large to very large, ranging from ω^2^p = 0.078 (Performance) to ω^2^p = 0.539 (UE). For IE Total, the interaction alone explained 49.1% of the variance using the bias-corrected estimator (ω^2^p = 0.491), confirming that the divergent temporal trajectory between groups, rather than time or group membership alone, accounts for a substantial proportion of outcome variance. Main effects of Time were significant for only SK, AOT, and Performance, indicating that for the remaining outcomes, temporal change was entirely conditioned on group membership.

### 3.6. Correlations Between Gain Scores

**Figure 2:**
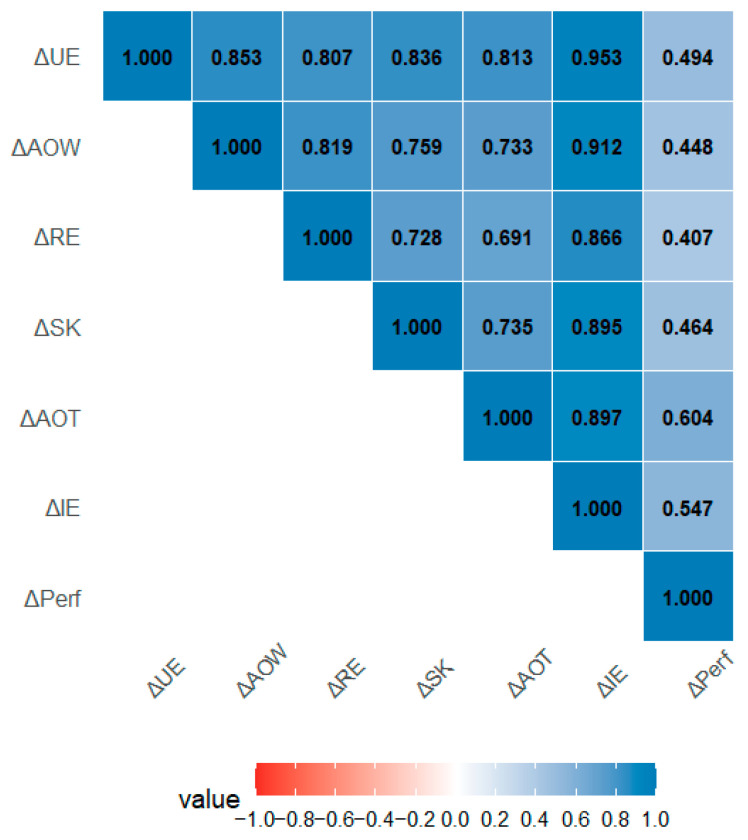
Pearson correlations between gain scores (Δ T2 − T1, *N* = 33) for the five emotional intelligence subscales (ΔUE, ΔAOW, ΔRE, ΔSK, ΔAOT), total EI (ΔIE), and shooting performance (ΔPerformance).

Pearson and Spearman correlations between gain scores are presented in Figure 2. Inter-subscale correlations among the five EI dimensions were uniformly large (r = 0.691–0.953, all *p* < 0.001), indicating strong co-variation across all EI dimensions at the level of gain scores. The correlation between IE Total and Performance was statistically significant and of moderate-to-large magnitude (r(31) = 0.517, *p* = 0.002; ρ = 0.535, *p* = 0.001). Among subscales, AOT showed the strongest association with Performance (r(31) = 0.504, *p* = 0.003; ρ = 0.547, *p* = 0.001), followed by UE (r = 0.489, *p* = 0.004) and RE (r = 0.476, *p* = 0.005). These associations were consistent across both Pearson and Spearman estimates, indicating robustness to distributional assumptions.

### 3.7. Regression Models

Two simple linear regression models were tested (Table 6). Both models yielded VIF = 1.000 and large Cohen’s *f* values (>0.35). Model A indicated that IE Total gain scores significantly predicted Performance gain scores (*β* = 0.517, *t*(31) = 3.359, *p* = 0.002, *R*^2^ = 0.267, *f* = 0.364), with each 10-point gain in IE Total associated with an additional 0.32-point gain in shooting performance. Model B indicated that post-test IE Total accounted for 43.7% of the variance in post-test shooting performance (*β* = 0.661, *t*(31) = 4.908, *p* < 0.001, *R*^2^ = 0.437, *f* = 0.777).

## 4. Discussion

### 4.1. Pre-Session Video Modeling as an EI Development Tool

The central and novel contribution of this RCT is providing preliminary evidence suggesting that a simple pre-session exposure to an anonymous athlete demonstrating the 9 m handball shooting technique; the sole difference between otherwise identical conditions was associated with significant improvements, with large observed effect sizes across all five EI dimensions and total EI in U14 novice players. To the best of current knowledge, this constitute one of the first controlled experimental investigations, that showed video observational modeling enhances EI in an athletic population [11].

Three theoretically grounded mechanisms have been proposed to potentially account for this type of effect, though none can be directly verified within the present design. First, attentional priming: pre-session video viewing directs participants’ attention toward performance-relevant emotional cues embedded in the footage the demonstrator’s composure after missed shots, sustained effort, and modulated arousal creating an emotional perceptual template participants subsequently apply during live practice [27,28,29,30,37]. Second, emotional schema acquisition: repeated across 18 sessions, systematic exposure to an athlete successfully navigating emotionally demanding performance situations progressively consolidates internal representations of adaptive emotional functioning, strengthening UE, RE, and AOT competencies [23,39]. Third, regulatory modeling: the demonstrator’s visible behavioral responses to performance outcomes provide concrete, observable templates for emotion regulation strategies that novice athletes have not yet developed through competitive experience alone [5].

The anonymous character of the model does not diminish but may in fact facilitate the learning conditions: without identity-based social comparison processes, participants can focus purely on the emotional and technical information conveyed by the footage [35,36]. The near-identical training conditions for both groups substantially reduce, though do not fully eliminate, alternative explanations; video viewing remains the most distinguishing feature between conditions. However, the relative contribution of these mechanisms cannot be disentangled within the present design. Several alternative explanations for the observed EI improvements must, however, be explicitly considered. First, participants in the VOM group may have experienced heightened motivation or engagement simply due to the novelty of the pre-session viewing component, independently of its emotional content a novelty effect that would be expected to attenuate over time. Second, the increased attentional focus induced by pre-session viewing may have improved performance by reducing cognitive load and heightening task readiness, rather than through genuine EI development. Third, social facilitation effects arising from the collective, group-based nature of the viewing sessions cannot be excluded as a contributing factor to the EI and performance improvements observed. Finally, given the exclusive use of self-report EI measures, improvements may partially reflect increased self-awareness or self-presentation effects rather than changes in actual emotional competence. The present design does not allow these explanations to be formally distinguished from the theorized mechanisms, and they represent important targets for future research.

### 4.2. Regression of EI in the Control Group

The significant regression of the CG on five of seven EI outcomes represents an empirical pattern that warrants cautious interpretation rather than a direct inference about the effects of standard training. Although the decline emerged under otherwise equivalent training conditions, it cannot be attributed to the control condition itself with confidence [21]. Multiple non-training explanations are equally plausible: regression to the mean following relatively high baseline scores in a small sample, self-report response fatigue at post-test, natural within-season fluctuation in adolescent self-perceptions of emotional competence, or differential expectancy effects between groups [55]. Accordingly, the bidirectional divergence observed here should be treated as an open descriptive finding rather than indirect evidence that standard training impairs EI development. The absence of a third measurement point and the exclusive reliance on a single self-report instrument preclude a definitive interpretation of this trajectory [17,18].

### 4.3. Performance Improvements and the EI-to-Performance Pathway

Shooting performance improved significantly in VOM and remained unchanged in CG, yielding a significant large inter-group effect. These performance gains are consistent with the well-established effects of pre-session video modeling on motor skill acquisition [24,25,26,27,32,33,34], where observation–practice coupling accelerates technical schema formation and stabilization.

The significant association between EI gains and performance gains provides original correlational evidence consistent with a co-development hypothesis, whereby EI and performance may both respond to the same VOM stimulus without one necessarily causing the other. It must be explicitly stated that the regression analyses reported in Section 3.7 establish statistical co-variation only they do not establish the direction, mechanism, or causal status of the EI–performance relationship. The present design, a single-site RCT without mediation analysis, without temporal separation of predictor and outcome, and relying exclusively on self-report EI measures, does not permit causal inference. Establishing whether EI changes precede, follow, or simply co-occur with performance changes would require longitudinal designs with multiple measurement points, or experimental designs in which EI is manipulated independently of the performance context. Model B demonstrates that EI level at post-test accounts for nearly 43.7% of variance in shooting accuracy, a relatively large proportion of variance than previously reported cross-sectional associations in handball [7,8,56]. This elevated association likely reflects the intervention-induced co-variation between EI and performance captured in the present longitudinal design, rather than a stable cross-sectional relationship. It is plausible, though speculative and not directly testable within the present design, that athletes who developed stronger AOT competencies may have concurrently enhanced their capacity to read defensive opponent cues during live shooting situations, potentially contributing to performance advantages beyond biomechanical improvement. This interpretation should be treated as a hypothesis for future investigation rather than a finding supported by the present data.

The finding that ΔAOT shows the strongest subscale–performance correlation is theoretically salient: in handball, shooting decisions against an active goalkeeper depend critically on the capacity to read defensive emotional and postural cues, anticipate goalkeeper reactions, and exploit moments of attentional lapses [15,16]. It is plausible, though speculative and not directly testable within the present design, that athletes who developed stronger AOT competencies through repeated video observation may have concurrently enhanced their in-game attentional reading of opponent cues, potentially contributing to shooting advantages beyond purely biomechanical improvement. This interpretation should be treated as a hypothesis for future investigation rather than an established finding.

The between-group effect sizes reported particularly for UE (*d* = 3.08) and IE Total (*d* = 2.80)—substantially exceed benchmarks from comparable sport psychology intervention research, where meta-analytic reviews report median between-group effect sizes of *d* = 0.40–0.70 for behavioral sport interventions [21,57], and *d* = 0.30–0.80 for EI training in athletic and educational contexts. Four design-specific mechanisms are likely to contribute to this inflation. First, Cohen’s d is sensitive to sample size: in small samples (*N* = 33), even modest mean differences relative to pooled standard deviations can produce large d values. Second, the bidirectional nature of group divergence the VOM group improving while the CG declined mechanically doubles the between-group numerator without necessarily reflecting the magnitude of treatment benefit on either group individually. Third, the exclusive use of self-report EI in an unblinded intervention context creates conditions for expectancy inflation and social desirability responding, which may have systematically elevated VOM scores and/or depressed CG scores. Fourth, the narrow five-shot performance proxy produces high within-cell variance in small samples, which can amplify apparent between-group differences. Taken together, these factors suggest that the reported effect sizes represent upper-bound estimates that are likely to attenuate substantially upon replication in larger and more heterogeneous samples. All practical recommendations derived from these findings must be explicitly conditioned on successful replication.

### 4.4. Validity and Sensitivity of the A-EIS

This trial provides preliminary evidence that the A-EIS is responsive to change in a controlled intervention context [45]. The instrument detected marked score shifts across groups, suggesting sensitivity to intervention-related variation; however, the clustered post-test distribution and low variance observed in the VOM group also raise the possibility of measurement compression, ceiling tendencies, or response-style effects. Accordingly, the present findings support the potential utility of the A-EIS in longitudinal sport research, but they should not be interpreted as definitive evidence of broad or systemic EI development without convergent behavioral or observer-based measures [3,4].

### 4.5. Limitations

Several limitations must be acknowledged. First, the sample is exclusively male, single-club, and Tunisian; generalization to female athletes, mixed samples, or other Arabic-speaking national contexts requires replication.

Second, and importantly, EI was measured only through self-report. Tools like the A-EIS are useful, but they come with well-known limitations. People may answer in socially desirable ways. They may also respond based on what they think the study expects from them. This is especially likely in intervention studies, where participants know which group they are in. Expectancy effects may also play a role. For example, participants in the VOM group may have expected to improve and adjusted their answers accordingly. These issues are particularly relevant here for several reasons. First, participants were not blinded to group allocation. Second, the intervention was new and positively framed, which may have encouraged more favorable responses. Third, the post-test EI scores in the VOM group showed a near-ceiling pattern, with very little variation between individuals. This could reflect real improvement, but it is also consistent with social desirability bias. Because no behavioral, observer-based, or performance measures were included, these alternative explanations cannot be ruled out. Future studies should include at least one non-self-report measure of EI. Examples include observer ratings of emotional behavior, coach evaluations of emotional regulation, physiological indicators such as heart rate variability under stress, or reaction-time tasks assessing emotional recognition. This would help clarify whether the reported improvements reflect actual change.

Third, the absence of a follow-up beyond T2 precludes conclusions about the maintenance or decay of EI and performance gains following intervention cessation. Fourth, the absence of assessor blinding for shooting performance not feasible in field conditions introduces a potential assessment bias that future trials should address through video-coded blind scoring. Fifth, the relatively small sample size may inflate effect size estimates, and replication with larger samples is necessary to confirm the magnitude and stability of the observed effects. Additionally, expectancy, attention, and novelty effects cannot be disentangled from the observed intervention effects within the present design. Participants in the VOM group may have experienced increased motivation, heightened task readiness, or more favorable self-evaluation simply because they received a structured and novel pre-session activity. These influences could plausibly account for a substantial part of the observed differences in both EI and performance and therefore limit strong inferences regarding the specific emotional or technical mechanisms of VOM.

### 4.6. Strengths, Practical Applications, and Future Directions

This RCT has several methodological strengths. The randomized, parallel-group design, together with allocation concealment and blinded outcome assessment, reduces both selection and detection bias. The within-session equivalence design in which both groups received identical training content, strengthens internal validity and helps isolate the pre-session VOM component as the active factor. The consistency between parametric and non-parametric tests across all seven outcomes further supports the robustness of the findings. In addition, reporting both η^2^p and the bias-corrected ω^2^p provides more precise estimates of explained variance in a small sample. Finally, this study offers the first experimental evidence that the A-EIS can function as a sensitive outcome measure in an RCT, contributing to measurement research in Arabic-speaking sport populations.

The findings also have practical relevance for coaching in youth handball and team sports more broadly. A short, standardized video (3–5 min), viewed collectively before training, is a low-cost and time-efficient tool that is easy to implement. Coaches do not need specialized psychological training. It is sufficient to select a technically accurate and emotionally informative video showing a representative athlete performing the target skill. The results suggest that EI components, especially emotion regulation and the ability to read others’ emotions, may develop alongside motor skills under VOM conditions. Based on this, coaches may benefit from integrating structured observational elements into regular training, even at early developmental stages (U12–U14). Sport federations and academies could also consider including pre-session video modeling as a practical complement to existing technical and physical training programs.

Several directions for future research emerge. Replication with female athletes, mixed-gender samples, and larger cohorts is needed to strengthen external validity. In addition, future studies should include follow-up assessments (e.g., 4–8 weeks after the intervention) to examine whether gains in EI and performance are maintained once VOM is no longer used. The absence of an active or placebo control condition such as exposure to neutral, non-emotional video content unrelated to the task constitutes a notable design limitation. Participants in the VOM group may have experienced increased motivation, heightened task engagement, greater attentional readiness, or expectancy effects simply because they received a structured and novel pre-session activity, independently of the emotional or technical content of the video. These influences could plausibly explain a substantial part of the observed group differences. Because they cannot be disentangled from the theorized observational learning effects within the present design, any interpretation that attributes the findings specifically to EI-related modeling processes must remain provisional. Future studies should therefore include an active control condition explicitly designed to separate observational-learning effects from novelty, attention, and expectancy influences. In addition, incorporating neurophysiological or behavioral measures (e.g., heart rate variability, attention tracking, observer ratings) alongside self-report EI instruments would strengthen construct validity and allow for a clearer understanding of the underlying mechanisms. Further work should also examine whether VOM effects on EI depend on characteristics of the model (e.g., novice vs. expert; peer vs. adult) or on specific features of the video content, such as emotional expressivity or the inclusion of failure scenarios. Addressing these factors would help refine the theoretical framework linking observational learning to emotional development.

## 5. Conclusions

This randomized controlled trial provides preliminary evidence that a brief pre-session video observational modeling intervention is associated with improvements in both emotional intelligence (across all five subscales) and 9 m shooting performance in U14 male handball players, beyond standard training alone. Improvements in EI were significantly associated with performance gains, and post-test EI was associated with a substantial proportion of variance in post-test shooting accuracy. These findings suggest that emotional processes may contribute to motor skill acquisition in youth sport. However, the causal direction and underlying mechanisms of this relationship cannot be determined from the present data.

From an applied perspective, integrating short, standardized pre-session video modeling into youth training programs appears to be a scalable and low-cost strategy to be a scalable and low-cost strategy with potential to support both psychological and technical development, although the specific mechanisms responsible for the observed benefits remain uncertain. The A-EIS also demonstrates good sensitivity as an outcome measure in controlled intervention settings.

Further research in larger, more diverse, and mixed-gender samples, combined with prospective registration and follow-up designs, is needed to confirm the magnitude, generalizability, and durability of these effects.

## Figures and Tables

**Table 1:** Anthropometric characteristics by group.

Variable	CG (*n* = 16) *M* (*SD*)	*t*(31)	*p*	VOM (*n* = 17) *M* (*SD*)
Height (cm)	162.88 (3.10)	0.40	0.692	163.29 (2.64)
Weight (kg)	53.88 (3.14)	0.05	0.961	53.94 (2.41)

Note. Values are presented as mean (*SD*). CG = control group; VOM = video observational modeling group. No significant between-group differences (*p* > 0.05).

**Table 2:** Descriptive statistics by group and time (*N* = 33).

Variable	VOM T1 *M* (*SD*)	95% CI	VOM T2 *M* (*SD*)	95% CI	CG T1 *M* (*SD*)	CG T2 *M* (*SD*)
UE	13.11 (3.95)	[11.2, 15.0]	19.16 (0.83)	[18.7, 19.6]	13.75 (1.43)	8.53 (2.62)
AOW	10.24 (3.33)	[8.5, 12.0]	14.71 (0.47)	[14.5, 14.9]	9.42 (2.58)	6.61 (2.77)
RE	7.47 (3.00)	[6.0, 8.9]	9.81 (0.42)	[9.6, 10.0]	7.44 (1.75)	3.75 (1.53)
SK	9.48 (3.46)	[7.7, 11.2]	14.69 (0.49)	[14.5, 14.9]	9.56 (3.41)	6.84 (2.51)
AOT	12.49 (4.33)	[10.3, 14.7]	19.58 (0.52)	[19.3, 19.8]	12.19 (3.56)	10.08 (4.23)
IE Total	52.93 (14.60)	[45.4, 60.4]	77.81 (1.48)	[77.0, 78.6]	52.31 (8.53)	35.72 (11.70)
Perf/5	2.53 (1.12)	[1.9, 3.1]	4.35 (0.70)	[4.0, 4.7]	2.64 (1.11)	3.17 (1.07)

Note. Values are presented as mean (*SD*). 95% CI = 95% confidence interval (*M* ± 1.96 × SE). T1 = pre-test; T2 = post-test. UE = Using Emotions; AOW = Appraisal of Own Emotions; RE = Regulation of Emotions; SK = Social Skills; AOT = Appraisal of Others’ Emotions; IE Total = total emotional intelligence score; Perf/5 = successful shots per five attempts.

**Table 3:** Paired *t*-tests and effect sizes—within-group pre/post evolution.

Variable	Δ*M* VOM	*t*(16)	*p*	*d*	95% CI *d*	Δ*M* CG	*t*(15)	*p*	*d*	95% CI *d*
UE	+6.06	6.709	<0.001	1.63	[0.90, 2.36]	−5.22	−5.812	<0.001	−1.45	[−2.15, −0.75]
AOW	+4.47	5.209	<0.001	1.26	[0.62, 1.90]	−2.81	−2.640	0.019	−0.66	[−1.20, −0.12]
RE	+2.34	3.286	0.005	0.80	[0.25, 1.35]	−3.69	−8.668	<0.001	−2.17	[−3.07, −1.27]
SK	+5.21	6.028	<0.001	1.46	[0.78, 2.14]	−2.72	−2.796	0.014	−0.70	[−1.25, −0.15]
AOT	+7.08	6.690	<0.001	1.62	[0.90, 2.34]	−2.11	−1.434	0.172	−0.36	[−0.87, 0.15]
IE Total	+24.88	7.197	<0.001	1.74	[0.99, 2.49]	−16.59	−4.313	<0.001	−1.08	[−1.70, −0.46]
Perf/5	+1.82	4.980	<0.001	1.21	[0.58, 1.84]	+0.53	1.552	0.141	+0.39	[−0.12, 0.90]

Note. Δ*M* = *M*T2 − *M*T1. Values are based on paired *t*-tests (VOM: *df* = 16; CG: *df* = 15). *d* = Cohen’s *d* for paired samples (Δ*M*/*SD* of differences); 95% CI computed using the formula *d* ± 1.96 × √(1/*n* + *d*^2^/2*n*). Negative *d* values in CG indicate deterioration from pre- to post-test. VOM = video observational modeling group; CG = control group. UE = Using Emotions; AOW = Appraisal of Own Emotions; RE = Regulation of Emotions; SK = Social Skills; AOT = Appraisal of Others’ Emotions; IE Total = total emotional intelligence score; Perf/5 = successful shots per five attempts. |*d*| ≥ 0.80 indicates a large effect.

**Table 4:** Comparison of gain scores (Δ T2 − T1): VOM vs. CG—parametric and non-parametric tests.

Variable	Δ*M* VOM (*SD*)	Δ*M* CG (*SD*)	*t*(31)	*p*	*U*	*p*	*d*	95% CI *d*	*r*
UE	+6.06 (3.72)	−5.22 (3.59)	8.846	<0.001	269.0	<0.001	3.08	[2.05, 4.11]	0.978
AOW	+4.47 (3.54)	−2.81 (4.26)	5.355	<0.001	248.5	<0.001	1.86	[1.04, 2.68]	0.827
RE	+2.34 (2.94)	−3.69 (1.70)	7.153	<0.001	272.0	<0.001	2.51	[1.58, 3.44]	1.000
SK	+5.21 (3.56)	−2.72 (3.89)	6.111	<0.001	255.5	<0.001	2.13	[1.27, 2.99]	0.879
AOT	+7.08 (4.37)	−2.11 (5.88)	5.118	<0.001	238.0	<0.001	1.77	[0.96, 2.58]	0.750
IE Total	+24.88 (14.26)	−16.59 (15.39)	8.037	<0.001	268.0	<0.001	2.80	[1.82, 3.78]	0.971
Perf/5	+1.82 (1.51)	+0.53 (1.37)	2.570	0.015	203.5	0.014	0.90	[0.18, 1.62]	0.496

Note. Δ = T2 − T1. *d* = Cohen’s *d* (pooled *SD*); 95% CI computed using the formula *d* ± 1.96 × √((*n*_1_ + *n*_2_)/(*n*_1_ × *n*_2_) + *d*^2^/2(*n*_1_ + *n*_2_ − 2)), with *n*_1_ = 17 (VOM) and *n*_2_ = 16 (CG). *r* = rank-biserial correlation (effect size for Mann–Whitney *U* test). *U* = Mann–Whitney test statistic. VOM = video observational modeling group; CG = control group. UE = Using Emotions; AOW = Appraisal of Own Emotions; RE = Regulation of Emotions; SK = Social Skills; AOT = Appraisal of Others’ Emotions; IE Total = total emotional intelligence score; Perf/5 = successful shots per five attempts.

**Table 5:** Mixed ANOVA—main effects and interaction with η^2^p and ω^2^p.

Variable	*F* Group (1,31)	*p*	η^2^p	ω^2^p	*F* Time (1,31)	*p*	η^2^p	ω^2^p	*F* Interaction (1,31)	*p*	η^2^p	ω^2^p
UE	68.06	<0.001	0.687	0.670	0.86	0.361	0.027	−0.002	78.25	<0.001	0.716	0.539
AOW	63.81	<0.001	0.673	0.656	1.91	0.177	0.058	0.014	28.68	<0.001	0.481	0.295
RE	34.58	<0.001	0.527	0.504	1.91	0.177	0.058	0.014	51.17	<0.001	0.623	0.432
SK	30.84	<0.001	0.499	0.475	4.44	0.043	0.125	0.050	37.35	<0.001	0.546	0.355
AOT	34.94	<0.001	0.530	0.507	8.56	0.006	0.216	0.103	26.20	<0.001	0.458	0.276
IE Total	73.06	<0.001	0.702	0.686	3.43	0.074	0.099	0.035	64.60	<0.001	0.676	0.491
Perf/5	4.66	0.039	0.131	0.100	22.70	<0.001	0.423	0.247	6.61	0.015	0.176	0.078

Note. Mixed-design ANOVA with Group (between-subjects) and Time (within-subjects) factors. η^2^p = partial eta squared; ω^2^p = omega squared (less biased estimator for small samples). UE = Using Emotions; AOW = Appraisal of Own Emotions; RE = Regulation of Emotions; SK = Social Skills; AOT = Appraisal of Others’ Emotions; IE Total = total emotional intelligence score; Perf/5 = successful shots per five attempts. Effect size thresholds: ≥0.01 small, ≥0.06 medium, ≥0.14 large.

**Table 6:** Simple linear regression models.

Model	Predictor	*B*	*SE*	*β*	*t*	*p*	*R* ^2^	*R*^2^adj	*f* ^2^
A: ΔIE → ΔPerformance	ΔIE Total	0.032	0.009	0.517	3.359	0.002	0.267	0.243	0.364
B: IE(T2) → Perf(T2)	IE Total T2	0.031	0.006	0.661	4.908	<0.001	0.437	0.419	0.777

***Note.*** *B* = unstandardized coefficient; *β* = standardized coefficient; *SE* = standard error; *R*^2^ = coefficient of determination; *R*^2^adj = adjusted *R*^2^; *f*^2^ = Cohen’s effect size (≥0.35 large). ΔIE Total = change in total emotional intelligence; ΔPerformance = change in shooting performance (Perf/5). IE Total T2 = post-test emotional intelligence score; Perf(T2) = post-test performance.

## Data Availability

Data are presented within the article.
